# GSA JOURNALS PANEL WITH THE EDITORS-IN-CHIEF

**DOI:** 10.1093/geroni/igae098.0904

**Published:** 2024-12-31

**Authors:** Jessica Kelley, Lewis Lipsitz

**Affiliations:** Chair; Co-Chair

## Abstract

GSA has a large portfolio of journals, giving authors opportunities to publish research on aging, from cells to policy. In this panel session, the current Editors-in-Chief will present information about their respective journal, including the scope, areas of special interest, and upcoming calls for papers. Following their remarks, we will have a moderated panel session with tips for submitting work, matching your research to the best-fitting journal, and strategies for success in the review process. Ample time will be given to audience questions. Editors will include: Steven Albert, Innovation in Aging; Gustavo Duque, Journal of Gerontology: Biological Sciences; Joe Gaugler, The Gerontologist; Duke Han, Journal of Gerontology: Psychological Sciences; Jessica Kelley, Journal of Gerontology: Social Sciences; Michael Lepore, Public Policy and Aging Research; and Lewis Lipsitz, Journal of Gerontology: Medical Sciences.

